# Innovative Polymeric Coatings with Dual Antifouling and Light-Activated Bactericidal Functions for Urinary Catheter Applications

**DOI:** 10.3390/polym16212974

**Published:** 2024-10-24

**Authors:** Po-Hsun Chen, Guan-Hua Chen, Wei-Bor Tsai

**Affiliations:** Department of Chemical Engineering, National Taiwan University, No. 1, Sec. 4, Roosevelt Rd., Taipei 106, Taiwan; f10524114@ntu.edu.tw (P.-H.C.); benson87111@gmail.com (G.-H.C.)

**Keywords:** zwitterionic polymer, photodynamic, catheter-associated urinary tract infections, surface coating, light-activated bactericidal effect

## Abstract

Catheter-associated urinary tract infections (CAUTIs) present significant health risks in medical settings, necessitating innovative solutions to prevent bacterial colonization on catheter surfaces. This study introduces a novel polymeric coating with dual antifouling and light-activated bactericidal properties to enhance the bactericidal efficacy of urinary catheters. The coatings were synthesized using a one-step process involving pyrogallol chemistry to deposit a copolymer composed of zwitterionic sulfobetaine for antifouling and sodium copper chlorophyllin, a photosensitizer that generates reactive oxygen species under light exposure to effectively kill bacteria. We evaluated the antifouling properties, cytocompatibility, and bactericidal performance of the coatings under various light conditions. The results showed significant reductions in bacterial adhesion, with light activation further endowing the catheter with bactericidal effects. Additionally, light could be delivered through an optical fiber within the catheter lumen to target and kill bacteria. The innovative coating using light-activated bactericidal action offers a promising approach to preventing CAUTIs, representing a potential breakthrough in developing safer and more effective urinary catheters.

## 1. Introduction

When medical conditions prevent a patient from eliminating urine, pressure can build up in the urinary bladder, potentially leading to kidney failure. Urinary catheters are commonly employed to manage conditions such as urinary incontinence and retention, as well as following surgical procedures on the prostate or genital areas. It is estimated that 15% to 25% of hospital patients are catheterized at some point during their stay [1]. Despite the utility of catheters, catheter-associated urinary tract infections (CAUTIs) remain the most prevalent bacterial infections globally, affecting over a million individuals annually in the United States and Europe alone [2]. With appropriate preventive measures, as many as 69% of CAUTIs could be prevented [3].

Preventing CAUTIs can be achieved by coating urinary catheters with materials that resist bacterial adhesion or actively kill bacteria, or both. The development of bacterium-resistant coatings often involves the deposition of an antifouling layer designed to prevent nonspecific interactions with biological components, such as proteins and bacteria, thereby reducing bacterial attachment and preventing biofilm formation [4,5]. A common strategy to create antifouling surfaces involves decorating the substrates with nonfouling polymers, including ethylene glycol-based polymers [6,7], zwitterionic polymers [8,9,10], and hydroxyethyl or hydroxypropyl polymers [11]. These hydrophilic layers adsorb significant amounts of water, forming a barrier that reduces nonspecific interactions between biomaterials and biological systems [12,13].

Bactericidal coatings can be fabricated based on contact-killing [14,15], release-killing [16,17], and stimulus-activated killing mechanisms [4,18]. While substrates coated with contact-killing agents such as quaternary ammonium compounds are effective, they face the challenge of long-term biofouling from proteins, dead bacteria, and debris, which reduces their effectiveness over time [19,20]. Materials utilizing release-killing agents like antibiotics and silver nanoparticles may encounter significant issues, such as the development of antibiotic resistance or possible toxicity related to silver nanoparticles while preventing catheter-related infection [21,22,23]. Emerging innovative stimulus-activated bactericidal coatings have shown bactericidal properties in response to specific stimuli such as pH, temperature, and light exposure, effectively combating bacterial growth and biofilm formation [4]. These coatings offer several advantages, including enhanced antibacterial efficacy, targeted action, and long-term protection. For instance, materials impregnated with photosensitizers like toluidine blue O and TiO_2_ have demonstrated effective UV-stimulated bactericidal capabilities [24,25]. However, UV light itself has inherent bactericidal properties and poses a risk to human health, particularly to the eyes. On the other hand, porphyrin-type compounds, such as chlorophyllin, cationic porphyrins, and sodium copper chlorophyllin, are effective photosensitizers that generate reactive oxygen species (ROS) when exposed to visible light, such as blue or red light [26,27,28,29,30]. The ROS induced by these compounds cause irreversible oxidative damage to bacterial cell membranes and other essential biomolecules, including DNA and enzymes. Coating catheters with porphyrin-type compounds provides a flexible, effective, and safe approach to confer antibacterial properties.

Over the past decade, multifunctional antibacterial coatings that combine antifouling and bactericidal capabilities have gained great prominence, enhancing the antibacterial efficacy of biomedical devices [4,31,32]. Developing simple, substrate-adaptable coating technologies is pivotal for advancing effective antibacterial coatings to improve the prevention of CAUTIs. In the last two decades, a mussel-inspired polydopamine coating can be deposited on a wide variety of materials and the ad-layer can anchor antifouling and bactericidal materials [33,34,35]. Similar to polydopamine (PDA) coatings, various plant-derived compounds featuring catecholic or galloyl groups can form robust coatings on diverse substrates [36,37]. These polyphenolic compounds offer a significant cost advantage over dopamine. Upon oxidation, catecholic or galloyl groups transform into quinones or other complex structures and subsequently oligomerize into higher-molecular-weight molecules [38]. These molecules have an inherent affinity for various surfaces, facilitating their deposition [39]. Our laboratory developed a series of antifouling coatings by conjugating zwitterionic polymers through polymerization of a phenolic compound, pyrogallol (PG) [8,40,41]. PG polymerizes through oxidation under alkaline conditions [8], in the presence of oxidants [40], or through electro-activation [41], forming an adlayer of polyPG film on various materials, while simultaneously co-depositing copolymers of sulfobetaine methacrylate and aminoethyl methacrylate (pSBAE). The amino moieties of pSBAE can react with PG, allowing them to be immobilized on the polyPG film. These pSBAE coatings effectively inhibit protein adsorption and cell adhesion while also withstanding harsh sterilization conditions.

In this study, we extended our previous research to develop an antibacterial surface using a one-step coating process that applies a copolymer endowed with both antifouling and light-activated bactericidal functionalities, which aims to prevent bacterial contamination associated with CAUTIs. The copolymer comprises an antifouling component, sulfobetaine, and a light-activated bactericidal component, sodium copper chlorophyllin, which produces bactericidal reactive oxygen species upon light exposure. The copolymer was deposited into silicone tubes in conjunction with the deposition of pyrogallol. The antibacterial efficacy and biocompatibility of the copolymer-coated silicone tubes were evaluated. Additionally, light was delivered through an optical fiber within the catheter lumen to target and kill bacteria. The surface modification technique developed herein demonstrates potential for significantly reducing the incidence of CAUTIs.

## 2. Materials and Methods

### 2.1. Materials

Sulfobetaine methacrylate (SBMA) was purchased from Hopax Chems (Kaohsiung, Taiwan). Sodium copper chlorophyllin (CC), 2-aminoethyl methacrylate hydrochloride (AEMA), dimethyl sulfoxide (DMSO), pyrogallol (PG), 2-mercaptoethanol, thiazolyl blue tetrazolium bromide (MTT), paraformaldehyde, Triton X-100, albumin-fluorescein isothiocyanate conjugate (FITC-BSA), and imidazole molecular biology reagent (Imd) were purchased from Sigma-Aldrich (Burlington, MA, USA). 2,2′-Azobis-isobutyronitrile (AIBN) and agarose were purchased from UniRegion Biotech (Taipei, Taiwan). Dulbecco’s modified Eagle medium/high glucose (DMEM/HG) was purchased from Life Technologies (Carlsbad, CA, USA). Antibiotic–antimycotic solution was purchased from Hyclone Laboratories (Logan, UT, USA). Fetal bovine serum (FBS) was purchased from Peak Serum (Wellington, CO, USA). Lysogeny broth (LB) was purchased from Bioshop (Burlington, ON, Canada). 4′,6-Diamidino-2-phenylindole (DAPI) was purchased from Thermo-Fisher Scientific (Waltham, MA, USA). N,N-Dimethyl-4-nitroaniline (RNO) was purchased from Alfa Aesar (Ward Hill, MA, USA).

The cell culture medium consisted of DMEM/HG and 1% antibiotic–antimycotic solution, supplemented with 10% FBS. Polydimethylsiloxane (PDMS) was prepared by mixing 184 A silicone elastomer and 184 B silicone elastomer (Dow Chemical, Midland, MI, USA) with a volumetric ratio of 10/1 and then curing at 70 °C for 2 h. BioPharm Plus silicone tube with an inner diameter of 1.6 mm and an outer diameter of 4.8 mm was purchased from Avantor Masterflex (Radnor, PA, USA).

Two different light sources were used in this study. The first was a simple LED light bulb, assembled by a local electronics store, providing three color options: blue (450–470 nm), green (520–540 nm), and red (630–660 nm). Details of these light sources are provided in S6 of the Supplementary Materials. Unfortunately, due to the simplicity and low cost of the LED light sources, the store could not provide light sources with different wavelength ranges at the same power. The second light source, used for the optical fiber-guided green laser experiment, was a Class IV 532 nm Green Solid-State Laser (catalog number MGL-N-532-AOM), purchased from Changchun New Industries Opto-electronics Tech. Co., Ltd. (Changchun, China).

### 2.2. Synthesis and Characterization of pSB and pSBCC

A copolymer of SBMA and AEMA (pSB) was synthesized through free radical polymerization, following a previously established protocol [8]. Using the optimal molar ratio of SBMA/AEMA determined in our previous study [42], 4.5 mmol of SBMA and 0.45 mmol of AEMA were dissolved in 18 mL of deionized water. This solution was then combined with 0.045 mmol of AIBN dissolved in 2 mL of DMSO and degassed by purging with nitrogen gas. To synthesize a copolymer incorporating sodium copper chlorophyllin (pSBCC), 0.45 mmol of CC was added to the monomer mixture. The polymerization was carried out at 70 °C for 20 h, followed by dialysis against deionized water using a cellulose membrane with a molecular weight cut-off of 3500 Dalton. The final products were lyophilized and stored at room temperature. The structural characteristics of the copolymers were analyzed using ^1^H NMR spectroscopy (AVIII-500, Bruker, Billerica, MA, USA) and UV-visible spectroscopy (Cary 300nc, Agilent, Santa Clara, CA, USA). The molecular weights of the copolymers were determined by gel permeation chromatography (UV-2075 system, JASCO, Tokyo, Japan). The composition of CC in pSBCC was determined by measuring the absorbance at 400 nm, based on a calibration curve generated from a series of known CC concentrations.

Singlet oxygen (^1^O_2_) production from CC and pSBCC solutions under different light conditions was evaluated using the imidazole and RNO (Imd/RNO) method [43]. CC and pSBCC solutions were prepared at a final concentration of 200 μM based on CC molecules. The solutions were mixed with 50 μM Imd/RNO and exposed to blue (450–470 nm), green (520–540 nm), or red (630–660 nm) light at a distance of 7 cm for 1 h at 37 °C. Singlet oxygen production was evaluated by measuring the absorbance at 440 nm, the wavelength at which Imd/RNO absorbs. A CC or pSBCC solution containing Imd/RNO without light exposure served as the control.

### 2.3. Preparation and Characterization of pSB and pSBCC Coatings

PG was dissolved in 10x phosphate-buffered saline (PB, pH 7.4) and then mixed in an equal volume with either pSB or pSBCC, both dissolved in PB to achieve final concentrations of 8 mg/mL PG and 48 mg/mL copolymers. The substrates were immersed in this solution for 12 h at 45 °C under constant agitation, followed by rinsing with deionized water and air-drying.

Surface wettability was assessed by the static contact angles of 2 μL drops of deionized water at room temperature using a contact angle measurement system (FTA-125, First Ten Angstroms, Newark, CA, USA). A minimum of ten spots of each sample were measured.

Fourier Transform Infrared (FTIR) spectra of the coatings were obtained using an FTIR spectrometer (Spectrum 100, PerkinElmer, Waltham, MA, USA) in attenuated total reflectance mode. Measurements were conducted with eight scans in the wavenumber range of 1000 to 4000 cm^−1^.

### 2.4. Quantitative Analysis of FITC-BSA Adsorption on pSB-Coated Substrates

The adsorption of FITC-BSA to pSB-coated PDMS in 96-well tissue culture polypropylene plate was quantified according to surface-bound fluorescence. The substrates were incubated with 100 μL FITC-BSA solution (20 μg/mL in phosphate-buffered saline, PBS) at 37 °C for 4 h. After incubation, the substrates were rinsed with PBS to remove any loosely bound FITC-BSA. The fluorescence intensity of the adsorbed protein was measured using a microplate reader (Synergy H1, BioTek, Winooski, VT, USA) with excitation and emission wavelengths of 495 nm and 525 nm, respectively. The amount of protein adsorbed onto the substrates was determined using a calibration curve established with known concentrations of FITC-BSA.

### 2.5. Evaluation of L929 Cell Adhesion and Cytotoxicity of pSB-Coated Substrates

L929 mouse fibroblasts obtained from the Food Industry Research and Development Institute (Hsin-Chu, Taiwan) were used to evaluate the anti-cell adhesion efficacy and cytotoxicity of the pSB-coated substrates. For anti-cell adhesion experiment, L929 cells with a cell density of 2 × 10^4^ cells/cm^2^ were seeded on the wells of 48-well tissue culture polystyrene (TCPS) plates with or without coating of pSB. After 24 h incubation, unattached cells were rinsed away with PBS. The attached cells were fixed with 4% paraformaldehyde and imaged using an optical microscope (TS-100, Nikon, Tokyo, Japan). The numbers of the attached cells were counted from the images.

The cytotoxicity of pSB-coated silicone tubes was evaluated using the extract method in accordance with ISO 10993-5 [44]. Briefly, 1 mL of cell culture medium was incubated with a coated silicone tube at 37 °C for 24 h. The extract was then added to 96-well TCPS plates which were pre-seeded with L929 cells at 2 × 10^4^ cells/cm^2^ and incubated at 37 °C for another 24 h. After this incubation period, the extract was removed and 100 µL MTT (1 mg/mL) solution was added to each well and incubated for 4 h at 37 °C. The formed formazan crystals were dissolved in 100 µL DMSO for 15 min, and the absorbance of the solution was measured at 570 nm. The absorbance from each sample was normalized to that of the culture medium.

### 2.6. Assessment of Bacteria Adhesion to pSB or pSBCC Coated PDMS

Staphylococcus aureus (*S. aureus*, ATCC21351) was used for bacterial adhesion experiments. The bacterial concentration was determined by measuring the absorbance at 600 nm. PDMS substrates were first cured on 48-well polypropylene plates and then coated with either pSB or pSBCC. To access bacterial adhesion, 100 µL of a bacterial solution (1 × 10^4^ CFU/mL) was added to each sample and incubated for 6 h at 37 °C. The adherent bacteria were then fixed with 4% paraformaldehyde and subsequently treated with 1% Triton X-100 for an hour. The fixed bacteria were stained with DAPI and imaged using fluorescence microscopy (IX71 Inverted Microscope System, Olympus, Tokyo, Japan). The fluorescence of each sample was then quantified from each image by ImageJ software 1.8.0 and normalized by that of the pristine PDMS [45].

### 2.7. Evaluation of Bactericidal Effects Under Light Illumination

The bactericidal efficacy of CC upon light illumination was evaluated by adding 1 mL of 6 mM CC in 1 mL bacterial solution (1 × 10^8^ CFU/mL) followed by 2 h irradiation of blue (450–470 nm), green (520–540 nm), and red (630–660 nm) light at a distance of 10 cm. The bactericidal effect was then assessed using the CFU method. Briefly, after light exposure, 50 µL of bacterial solution was plated onto agar and spread evenly. The colony-forming units were counted after incubating at 37 °C for 20 h.

To evaluate the antibacterial efficacy of pSB and pSBCC coatings, 0.5 cm long tubes were immersed in 1.5 mL of bacterial solution containing either *S. aureus* or Escherichia coli (*E. coli*, ATCC23501) (1 × 10^4^ CFU/mL) and incubated for 4 h at 37 °C. Then, each tube was lightly rinsed with PBS. After rinsing, the samples were exposed to blue, green, or red light in PBS or LB for 2 h from the side. The attached bacteria were collected by sonication for 30 min, and viable bacteria were quantified using the CFU method.

In the experiment using green laser illumination inside silicone tube lumens, several silicone tubes, each 0.5 cm in length, were threaded with an optical fiber and placed in a bacterial solution. A green laser (532 nm; 1.5 W) was transmitted through the optical fiber into the lumens of the silicone tubes for 2 h at 37 °C. After exposure, the viable bacteria on the tubes were quantified using the CFU method.

### 2.8. Statistical Analysis

Statistical analyses between all groups were performed in GraphPad Prism 9.0 using the one-way or two-way ANOVA followed by Tukey’s multiple comparison test. *, **, and *** indicate *p* value < 0.05, 0.01, and 0.001, respectively. All the values are presented as mean ± standard deviation.

## 3. Results and Discussion

### 3.1. Light-Induced Bactericidal Properties of CC

**Figure 1:**
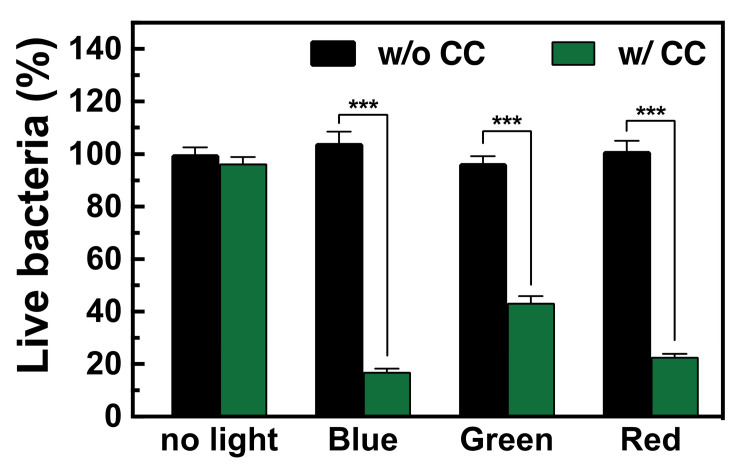
The ratios of the amounts of live bacteria after exposure to blue, green, or red light for 2 h (*n* = 4) with or without the presence of sodium copper chlorophyllin (CC). All data are shown as mean ± standard deviation, *** *p* < 0.001.

The bactericidal efficacy of sodium copper chlorophyllin (CC) was evaluated using the CFU method. CC was added to a bacterial solution and then exposed to different types of light. As shown in Figure 1, CC alone exhibited no bactericidal activity without light activation. Similarly, exposing bacterial solutions to blue, green, or red light without CC did not result in any bactericidal effects. However, when CC was activated by light, bacterial viability was significantly reduced. Specifically, bacterial viability decreased by 87.3 ± 2.20% under blue light, 53.2 ± 1.80% under green light, and 78.5 ± 2.10% under red light. These results indicate that CC has light-activated bactericidal properties. Therefore, CC was incorporated into the pSB copolymer for further evaluation. One thing to note is that the power levels of the different lights vary, so the photodynamic effects cannot be attributed solely to their wavelengths.

### 3.2. Characterization of pSB and pSBCC

CC contains unsaturated vinyl groups that can be incorporated into a polymer via free radical polymerization [46,47]. We synthesized copolymers of sulfobetaine methacrylate and aminoethyl methacrylate (pSB) or those with additional CC (pSBCC). Their compositions were evaluated using ^1^H NMR spectra (see S1 in Supplementary Materials). The peak at 3.76 ppm corresponds to the two protons adjacent to the quaternary amine of SBMA, while the peak at 4.24 ppm corresponds to the two protons adjacent to the oxygen atom of AEMA [8]. The molar ratios of SBMA to AEMA in pSB and pSBCC, determined by the ratio of the area of the peak at 3.76 ppm to that at 4.24 ppm, were 15.35 and 19.53, respectively.

However, we did not detect the characteristic peak of CC around 3.59 ppm in the spectrum of pSBCC, possibly due to the interference of the magnetic properties of copper ions and overlapping peaks with SBMA [46]. Therefore, the content of CC was estimated based on optical absorbance [46] (see S2 in Supplementary Materials). The absorbance of CC was observed approximately at 400 and 640 nm, which were also present in pSBCC. Assuming the absorbance of CC at 400 nm in its free form and in the copolymer is identical, 1 mg of pSBCC contained 0.0630 mg of CC (see S3 in Supplementary Materials). The molar ratio of SBMA, AEMA, and CC in pSBCC was then calculated to be 19.5:1.00:0.522. The molecular weights of pSB and pSBCC were estimated to be around 100 kDa using GPC.

### 3.3. Surface Characterization of pSB-Coated Substrates

**Figure 2:**
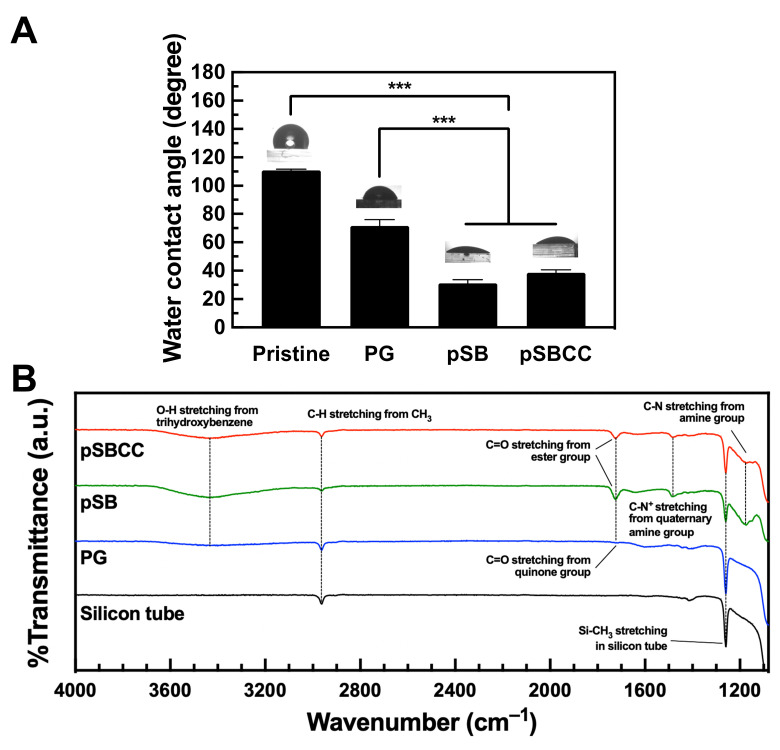
Surface characterization of pristine PDMS. Only pyrogallol (PG), the copolymer of SBMA and AEMA (pSB), and the copolymer incorporating sodium copper chlorophyllin (pSBCC) coatings were determined using (**A**) WCA measurements on the coatings on PDMS (*n* = 8) and (**B**) ATR-FTIR spectra of the silicon tubes coated with PG, pSB, and pSBCC. All data are shown as mean ± standard deviation. ***, *p* < 0.001 vs. Pristine.

The wettability of PDMS coated with pSB or pSBCC was evaluated using water contact angle (WCA) measurements. The WCA on pristine PDMS was 110.8°, while the pyrogallol (PG) coating reduced the WCA to 71.4°, indicating enhanced hydrophilicity due to the PG coating (Figure 2A) [40]. The addition of pSB or pSBCC further decreased the WCA to 31.1° and 38.4°, respectively.

The coatings of PG, pSB, and pSBCC on the silicon tubes were characterized by ATR-FTIR analysis (Figure 2B). The PG coating introduced a peak at 3427 cm^−1^ from O-H from phenol and C=O from quinone after the partial oxidation of PG at 1723 cm^−1^ on PDMS [48]. The addition of pSB generated peaks at 1725, 1482, and 1174 cm^−1^, corresponding to C=O, C-N^+^ from the quaternary amine group, and C-N stretch of pSB, respectively, while the characteristic peak of Si-CH_3_ significantly decreased. However, there was no obvious difference between the spectra of pSB and pSBCC, likely due to the relatively low content of CC in the copolymer. These results indicate that pSB or pSBCC was successfully conjugated onto the substrates via PG deposition.

### 3.4. Antifouling Efficacy of pSB and pSBCC Coated Surfaces

**Figure 3:**
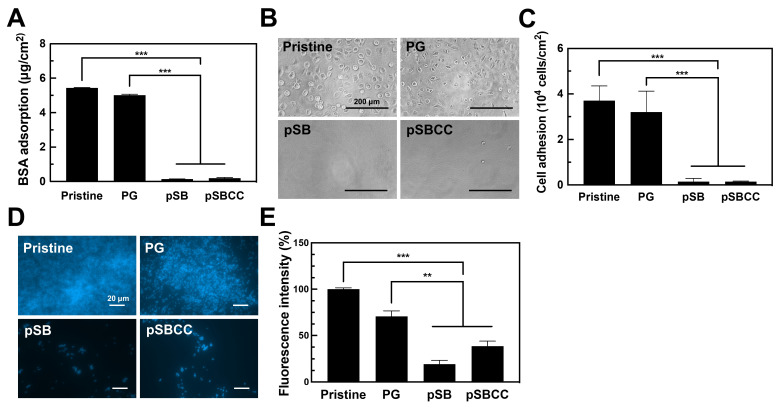
The evaluation of antifouling efficacy of the pyrogallol (PG), only the copolymer of SBMA and AEMA (pSB), and the copolymer incorporating sodium copper chlorophyllin (pSBCC) coatings: (**A**) the adsorption of FITC-BSA on the coated of PDMS (*n* = 4); (**B**) the microscope images of L929 cell adhesion on the coated TCPS after 24 h incubation; (**C**) the numbers of the cells adhered on the coated TCPS after 24 h incubation (*n* = 3); (**D**) the DAPI-stained fluorescent images of the adhering *S. aureus* on the coated PDMS after 6 h incubation; and (**E**) the quantification of fluorescence intensity from the images from D (*n* = 3). All data are shown as mean ± standard deviation, **, *p* < 0.01 and ***, *p* < 0.001 vs. Pristine.

Resisting protein adsorption is a critical aspect of antifouling. In this study, bovine serum albumin (BSA) was used to evaluate the protein resistance of modified surfaces. The adsorption of BSA was measured at 5.43 and 5.01 μg/cm^2^ for pristine and PG-coated PDMS, respectively (Figure 3A). Coating with pSB significantly reduced FITC-BSA adsorption to 0.141 μg/cm^2^, while the pSBCC coating also markedly decreased protein adsorption to 0.195 μg/cm^2^. Compared to pristine PDMS, BSA adsorption was reduced to approximately 3% on pSB-coated surfaces.

Resisting cell attachment is another crucial aspect of antifouling properties. The extent of cell adhesion on various coated TCPS plates was assessed. A substantial number of L929 cells adhered to both pristine and PG-coated TCPS plates, while pSB and pSBCC coatings demonstrated a significant reduction in cell attachment (Figure 3B). Quantitative analysis showed no significant difference in cell adhesion between PG-coated and pristine TCPS plates. However, cell attachment was reduced to approximately 4% on pSB and pSBCC coatings compared to pristine TCPS plates (Figure 3C).

For the bacteria adhesion test, *S. aureus* was seeded on PDMS with different coatings and incubated for 6 h. The adhering bacteria were stained with DAPI and observed under fluorescence microscopy (Figure 3D). Bacteria adhered confluently on both pristine and PG-coated PDMS. In contrast, pSB and pSBCC coatings showed a significant reduction in bacteria adhesion. The fluorescent intensities of DAPI on pristine PDMS, PG, pSB, and pSBCC coatings were quantified as 156, 111, 30.4, and 60.2 a.u., respectively. Then, the intensity was normalized to 71.2%, 19.5%, and 38.6% of the intensity on pristine PDMS, respectively (Figure 3E). These results are consistent with the antifouling effects observed in the protein and cell adhesion tests.

### 3.5. Cytotoxicity Assessment of pSB-Coated Silicone Tubes

**Figure 4:**
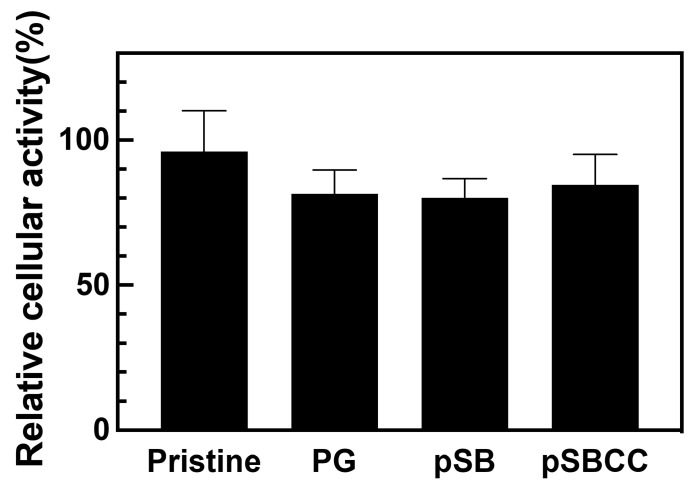
Cytotoxicity of the extracts of the silicone tubes coated with only pyrogallol (PG), the copolymer of SBMA and AEMA (pSB), and the copolymer incorporating sodium copper chlorophyllin (pSBCC) coatings using the MTT assay (*n* = 3). All data are shown as mean ± standard deviation. No significant difference appeared between the different groups.

Silicone tubes, uncoated and coated with pSB or pSBCC, were extracted with culture medium, and the cytotoxicity of the extracts was evaluated using the MTT assay. The relative cellular activities compared to those observed with the culture medium were 80.2% for pSB and 84.6% for pSBCC, as shown in Figure 4. These values were not significantly different from those for PG-coated samples, indicating that these coatings exhibited acceptable levels of cytotoxicity.

### 3.6. Bactericidal Efficacy of pSBCC Coating with Light Illumination

**Figure 5:**
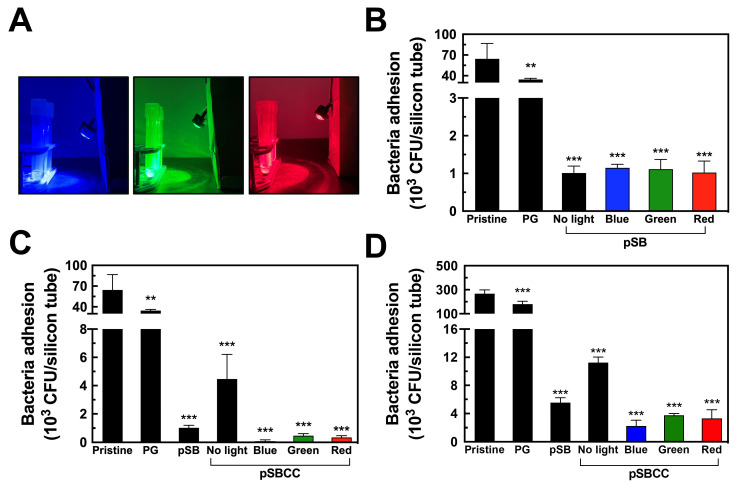
(**A**) The photos of the silicone tubes exposed to blue, green, and red light. (**B**) The numbers of bacteria on the copolymer of SBMA and AEMA (pSB) exposed to blue, green, and red light in PBS (*n* = 4). (**C**) The numbers of bacteria on the copolymer incorporating sodium copper chlorophyllin (pSBCC) coatings exposed to blue, green, and red light in PBS (*n* = 4). (**D**) The numbers of bacteria on the copolymer incorporating sodium copper chlorophyllin (pSBCC) coatings exposed to blue, green, and red light in bacterial LB (*n* = 3). Bacterial adhesion on the silicon tubes was quantified by the CFU method. All data are shown as mean ± standard deviation, **, *p* < 0.01 and ***, *p* < 0.001 vs. Pristine.

We evaluated the bactericidal efficacy of the pSBCC coating under light illumination. Silicone tubes were coated with PG, pSB, and pSBCC, and then exposed to a blue (450–470 nm), green (520–540 nm), and red (630–660 nm) light bulb for 2 h (Figure 5A). The PG coating reduced bacterial adhesion from 64,500 to 34,500 CFU per silicone tube, achieving a reduction ratio of 46.5% (Figure 5B). The pSB coating further decreased bacterial adhesion by 98.4%, but exposure to blue, green, or red light did not further enhance the resistance of pSB to bacterial adhesion. The results indicate that the pSB coating resists bacterial adhesion but does not have bactericidal ability.

On the other hand, pSBCC reduced the adhesion of *S. aureus* to 4467 CFU per silicone tube without light exposure, achieving a reduction ratio of 93.1%, slightly less effective than pSB of 98.4% (Figure 5C), which indicates that the addition of CC reduces the antifouling efficacy of pSB, as CC lacks antifouling properties. However, when exposed to blue, green, and red light, pSBCC demonstrated further reductions in bacterial adhesion to 90, 473, and 350 CFU per silicone tube, corresponding to reduction ratios of 99.86%, 99.27%, and 99.46%, respectively. Compared to the antifouling pSB surface (Figure 5B), the pSBCC surface possesses an additional light-activated bactericidal capacity, which effectively kills bacteria and reduces surface-bound bacterial contamination. In the case of the Gram-negative *E. coli*, the bactericidal efficacy upon exposure to blue, green, and red lights showed that pSBCC coatings reduced *E. coli* adhesion to 15, 3098, and 480 CFU per silicon tube, with reduction ratios of 99.93%, 85.0%, and 97.7%, respectively (see S4 in Supplementary Materials). The lower bactericidal effect on *E. coli* might be attributed to Gram-negative bacteria being less susceptible to ^1^O_2_ than Gram-positive bacteria [49]. These results indicate that the pSBCC coating possesses both antifouling and light-stimulated bactericidal efficacy.

Next, we tested the pSBCC coating immersed in bacterial LB, which supports bacterial growth. The number of bacteria on the pristine silicone tube increased to 267,000 CFU per silicone tube, roughly four times that in PBS (Figure 5D). The PG coating slightly decreased bacterial attachment to 181,500 CFU per silicone tube, achieving a reduction ratio of 32.0%. In comparison, bacterial attachment decreased to 5500 CFU per silicone tube on the pSB coating, achieving a reduction ratio of 97.9%, still significantly higher than in PBS. Interestingly, the bacterial attachment to pSBCC without light exposure was 11,250 CFU per silicone tube, resulting in a reduction ratio of 95.8%, less than that of pSB. The increase in adherent bacteria is likely due to the increased bacterial growth in LB and on the surfaces of the silicone tubes. Exposure of the pSBCC-coated tubes to blue, green, and red light reduced bacterial adhesion to 2250, 3750, and 3300 CFU per silicon tube, corresponding to reduction ratios of 99.16%, 98.60%, and 98.76%, respectively. These results demonstrate that the light-activated bactericidal efficacy of pSBCC significantly reduces bacterial contamination even in environments favorable to bacterial growth.

The bactericidal mechanism of CC in pSBCC-coated silicone tubes could result from the generation of reactive oxygen species (ROS) upon photodynamic activation [50]. Previous studies reported that CC, under visible-light irradiation, could generate various reactive oxygen species (ROS), including singlet oxygen ^1^O_2_ and hydroxyl radicals (·OH) [51,52]. However, these studies demonstrate that CC predominantly produces singlet oxygen when exposed to visible light. To investigate this, we measured the production of ^1^O_2_ in CC and pSBCC solutions under light irradiation. Singlet oxygen production in the CC solution was assessed by monitoring the bleaching of RNO, with absorbance reductions of 10.7%, 1.8%, and 4.5% under the exposure of blue, green, and red light, respectively (Figure S5A in Supplementary Materials). Similarly, the pSBCC solution showed absorbance reductions of 7.3%, 1.6%, and 3.7% under the exposure of blue, green, and red light, respectively (Figure S5B in Supplementary Materials). These results correspond with the absorption spectrum of CC, which has a major peak around 410 nm and a minor peak near 620 nm (Figure S2 in Supplementary Materials). These findings support the hypothesis that the bactericidal action of light-activated pSBCC is mediated by singlet oxygen generation.

### 3.7. Irradiation of Bacteria Through Optical Fiber-Guided Green Laser Illumination Inside Silicone Tubes

**Figure 6:**
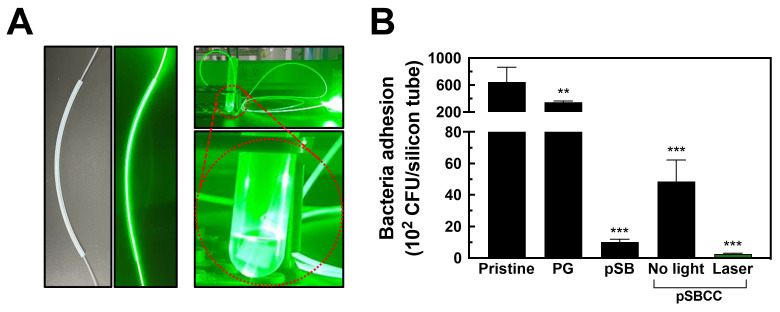
(**A**) Illustration of laser guidance in a silicon tube. A green laser was guided via an optical fiber for light-emitting from the lumen of the silicone tube. (**B**) The amount of bacteria adhesion on the silicone tubes coated with only pyrogallol (PG), the copolymer of SBMA and AEMA (pSB), and the copolymer incorporating sodium copper chlorophyllin (pSBCC) coatings (*n* = 4). All data are shown as mean ± standard deviation, **, *p* < 0.01 and ***, *p* < 0.001 vs. Pristine.

To demonstrate the potential clinical applicability of our system, an optical fiber was integrated within a silicone tube to irradiate bacterial contaminants. Although green light is the least effective in killing bacteria, we used green laser for this experiment because our laboratory only has access to a green laser source. Therefore, a green laser was directed into the lumen of a silicone tube via an optical fiber to facilitate in situ eradication of bacteria (Figure 6A). High levels of bacterial attachment were observed on both pristine and PG-coated tubes (Figure 6B), consistent with previous results, while bacterial adhesion was significantly reduced on pSB- and pSBCC-coated tubes to 1013 and 4872 CFU per silicon tube, achieving reduction ratios of 98.43% and 92.45%, respectively. Following exposure to the green laser, bacterial attachment further decreased to 260 CFU per silicone tube, achieving a reduction ratio of 99.60%. These results demonstrate the effectiveness of the light-induced bactericidal coating and underscore its potential clinical utility.

This study demonstrates that a copolymer with antifouling and light-activated bactericidal properties can be easily immobilized onto catheters through PG deposition. Laser light can be delivered into the lumen of a pSBCC-coated silicone tube via an optical fiber to achieve in situ bacterial eradication. However, there is potential for further improvement of this system. First, replacing the green laser with a blue laser could enhance bactericidal efficacy. Second, using a different type of chlorophyllin with higher photodynamic efficiency instead of CC could further improve performance. For instance, chlorin e6 and sodium magnesium chlorophyllin have been shown to produce twice the amount of singlet oxygen and more effectively kill bacteria compared to sodium copper chlorophyllin under the same irradiation conditions [53].

## 4. Conclusions

In this study, we successfully developed a novel polymeric coating with dual antifouling and light-activated bactericidal properties for potential application to urinary catheters. The copolymer, composed of sulfobetaine for antifouling and sodium copper chlorophyllin as a photosensitizer, was effectively synthesized and coated onto silicone tubes using pyrogallol chemistry. The antifouling properties demonstrated significant reductions in bacterial adhesion, while the light-activated bactericidal function further enhanced antimicrobial efficacy. Integrating an optical fiber within the silicone tube to deliver targeted light irradiation underscores the potential clinical utility of this system. This innovative approach offers a promising solution for preventing CAUTIs by combining robust antifouling properties with efficient, light-induced bactericidal action.

## Data Availability

Data are contained within the article or Supplementary Materials.

## Supplementary Materials

The following supporting information can be downloaded at https://www.mdpi.com/article/10.3390/polym16212974/s1, Figure S1: Characterization of pSB and pSBCC copolymers; Figure S2: UV-visible spectral analysis and quantification of CC in pSBCC; Figure S3: Determination of the concentration of CC in the pSBCC copolymer solution; Figure S4: Bactericidal efficacy of pSBCC coatings on *E. coli* with light illumination; Figure S5: Singlet oxygen production assessed by measuring the bleaching of RNO; Table S1: LED light sources.
