# Supplementary material for: Innovative Polymeric Coatings with Dual Antifouling and Light-Activated Bactericidal Functions for Urinary Catheter Applications
Source: Polymers (Basel). 2024 Oct 24;16(21):2974. doi: 10.3390/polym16212974 (PMC11548113; doi:10.3390/polym16212974)
Supplement: Supplementary file 1 [file polymers-16-02974-s001.zip › polymers-3237624-supplementary.pdf]

Supplementary Materials

S1. Characterization of pSB and pSBCC copolymers

The lyophilized samples were dissolved in D_2O for 1H NMR characterization. The peaks at 1.97 ppm (peak a), 1.09 ppm (peak b), 4.46 ppm (peak c), 3.76 ppm (peak d), 3.18 ppm (peak e), 3.55 ppm (peak f), 2.23 ppm (peak g), 2.94 ppm (peak h) correspond to the hydrogen in SBMA. The peaks at 1.97 ppm (peak a), 1.09 ppm (peak b), 4.24 ppm (peak i), and 3.30 ppm (peak j) correspond to the hydrogen in AEMA. The peak at 3.59 ppm (peak k) corresponds to the hydrogen in CC. The molar ratio of SBMA to AEMA in pSB and pSBCC, determined by the ratio of the area of the peak at 3.76 ppm (peak d) to that at 4.24 ppm (peak i), was 15.35 and 19.53, respectively. However, we did not detect the characteristic peak of CC around 3.59 ppm (peak k) in the spectrum of pSBCC, possibly due to the interference of the magnetic properties of copper ions and overlapping peaks with SBMA.

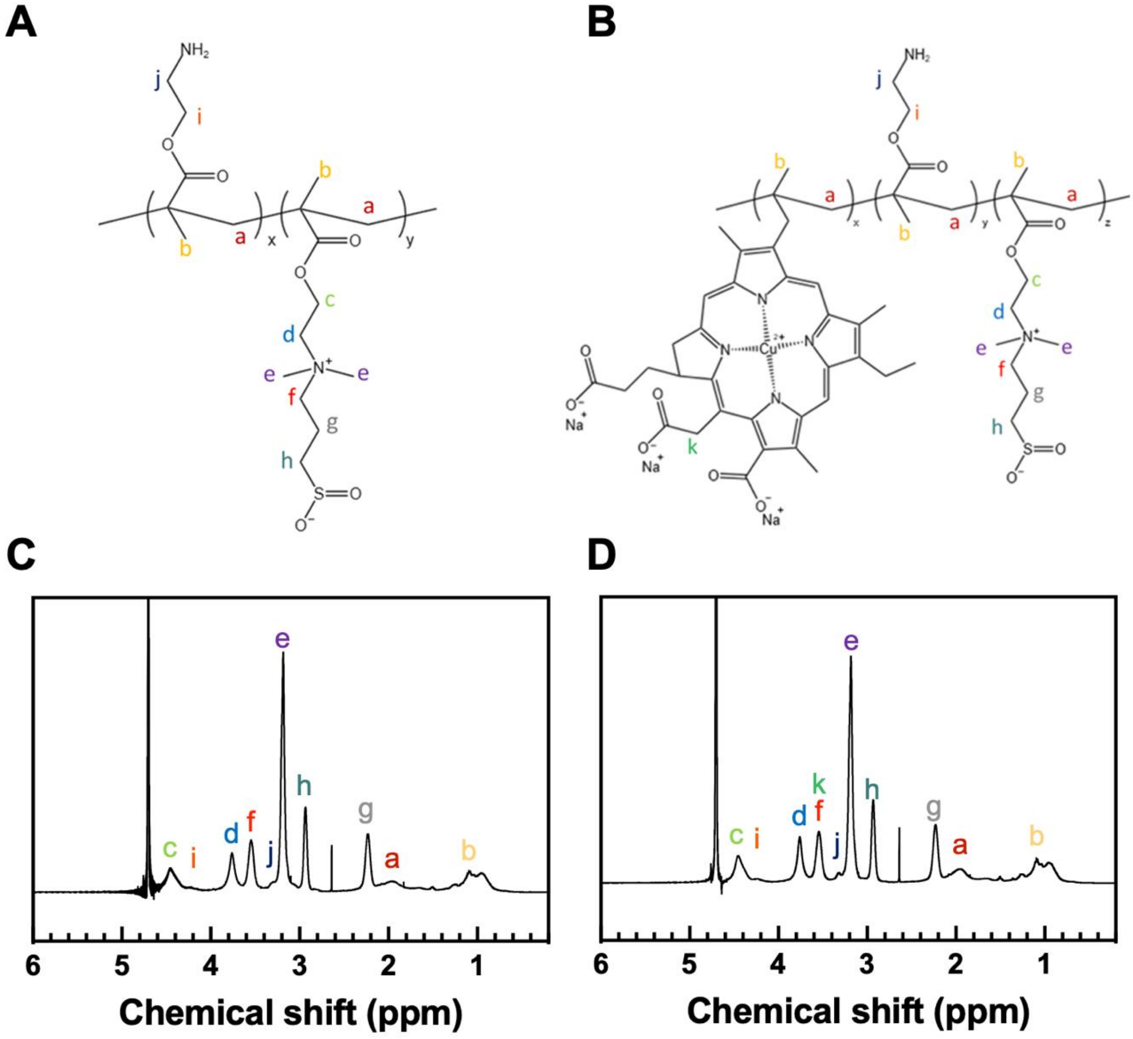

Figure S1. The structure and hydrogen positioning in (A) pSB and (B) pSBCC. Characterization from ^{1}H NMR spectra of (C) pSB (D) pSBCC.

S2. UV-visible Spectral Analysis and Quantification of CC in pSBCC

Lyophilized CC, pSB, and pSBCC were dissolved at concentrations of 0.07, 0.4, and 0.4 mg/mL, respectively, in 10x phosphate-buffer saline (PB, pH 7.4). The UV-visible spectra indicated that the maximum absorbance for CC and pSBCC occurred at approximately 400 nm, while pSB showed no significant absorbance at this wavelength. Additionally, a minor absorbance peak was observed at around 640 nm for both CC and pSBCC. Therefore, the amount of CC in pSBCC was further quantified based on OD_{400}.

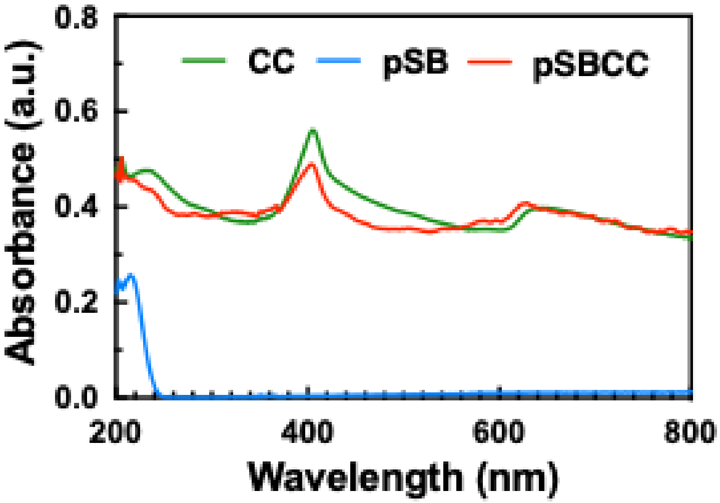

Figure S2. UV-visible spectra of CC, pSB, and pSBCC in 10x phosphate-buffer saline (PB, pH 7.4) measured from 200 to 800 nm.

S3. Determination of the concentration of CC in the pSBCC copolymer solution

The CC concentration in pSBCC was obtained from the absorbance at 400 nm. The concentration of CC was then calculated by the following equation (1).

$$OD_{400}\text{=Concentration of CC in pSBCC solution } \left(\frac{\mu g}{mL}\right) \times 0.01143 + 0.008865 \text{ -- eq.(1)}$$

The extinction coefficient calculated from Figure S3 is 828 m^2/mol.

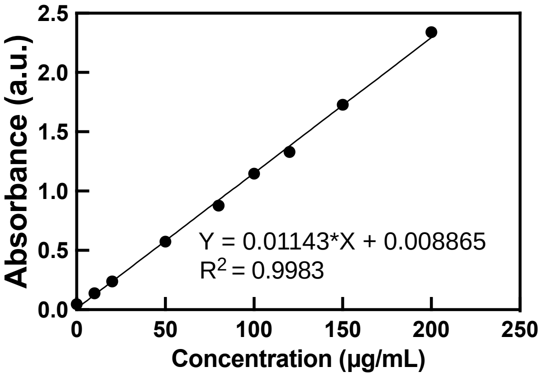

Figure S3. Standard curve of concentration of CC. The determination of the CC amount per mg of pSBCC ($n = 4$).

S4. Bactericidal efficacy of pSBCC coatings on *E. coli* with light illumination

A gram-negative bacteria evaluation using Escherichia coli (*E. coli*, ATCC23501) was conducted with varied coatings alongside a parallel assessment with *S. aureus*. Briefly, PG was dissolved in PB solution in an equal volume of pSB or pSBCC to achieve final concentrations of 8 mg/mL PG and 48 mg/mL copolymers. The 0.5 cm-long silicon tubes were immersed in the solution for 12 hours at 45°C under constant agitation, followed by rinsing with deionized water and air-drying. Then, tubes were immersed in 1.5 mL of bacterial solution containing *E. coli* (1×10^4 CFU/mL) and incubated for 4 hours at 37 °C. Then, each tube was lightly rinsed with PBS. After rinsing, the samples were exposed to blue (450-470 nm), green (520-540 nm), or red light (630-660 nm) from the side with a distance of 10 cm in PBS for 2 hours. The attached bacterial was collected by sonication for 30 minutes, and viable bacteria were quantified using the CFU method.

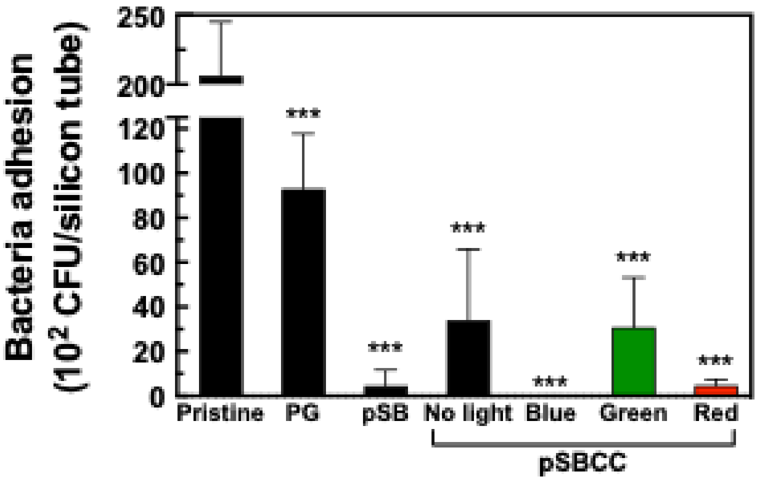

Figure S4. The numbers of *E. coli* on pSBCC exposed to blue, green, and red light in PBS ($n = 6$). The bacterium adhesion on the silicon tubes was quantified by the CFU method. All data are shown as mean ± standard deviation, ***, $p < 0.001$ vs. Pristine.

S5. Singlet oxygen production assessed by measuring the bleaching of RNO

Singlet oxygen production from CC and pSBCC under various light conditions was assessed using the imidazole and RNO (Imd/RNO) method. CC and pSBCC solution were prepared at a final concentration of 200 µM based on CC molecules. The solutions were mixed with 50 µM Imd/RNO and exposed to blue (450-470 nm), green (520-540 nm), or red (630-660 nm) light with a distance of 7 cm for an hour incubated at 37°C. Singlet oxygen production was then assessed by measuring the absorbance at 440 nm. The absorbance of pure CC and pSBCC was zeroed before measurement to isolate the

absorbance from RNO. The consumption of RNO under each light condition was calculated relative to the dark control, indicating the relative amount of singlet oxygen produced by both CC and pSBCC.

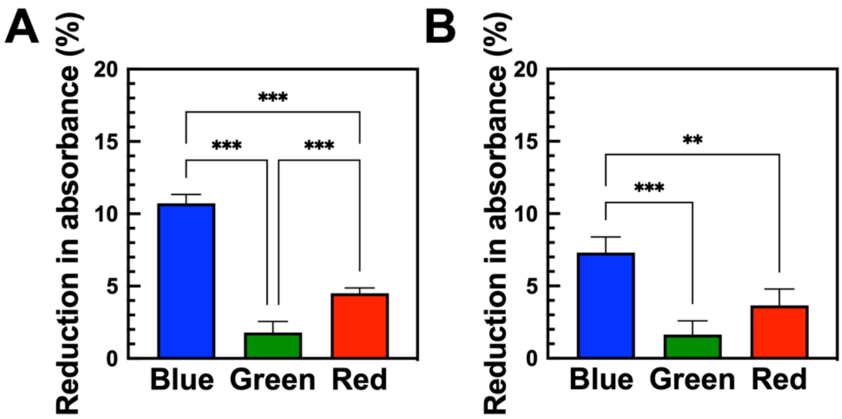

Figure S5. The consumption of RNO by singlet oxygen under light illumination was measured from the reduction in absorbance at 440 nm for (A) CC and (B) pSBCC at equivalent molarity to CC (n = 4). All data are shown as mean ± standard deviation, **, p < 0.01 and ***, p < 0.001.

S6. LED light sources

The light sources used were simple LED bulbs connected to a circuit, as shown on the right. A local electronics store customized these bulbs to provide specific colors: blue (450-470 nm), green (520-540 nm), and red (630-660 nm). As shown in the table below, the light intensity was measured by a power meter (COHERENT FieldMax$_{II}$, PA, USA).

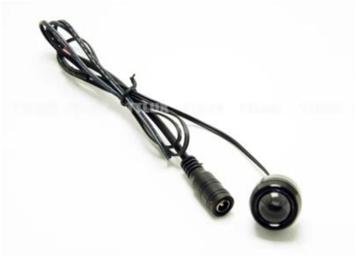

LED light	Light intensity (mW/cm^2)	
	Distance at 7 cm	Distance at 10 cm
Blue	7.1	3.7
Green	1.6	0.8
Red	1.9	0.9

Table S1. The light intensity of blue, green and red LED light bulbs measured from a light of 7 cm and 10 cm.
